# Supplementary material for: Transport and Permeation Properties of Dapivirine: Understanding Potential Drug-Drug Interactions
Source: Pharmaceutics. 2022 Sep 14;14(9):1948. doi: 10.3390/pharmaceutics14091948 (PMC9501983; doi:10.3390/pharmaceutics14091948)
Supplement: Supplementary file 1 [file pharmaceutics-14-01948-s001.zip › pharmaceutics-1875313-supplementary.pdf]

Supplementary Materials

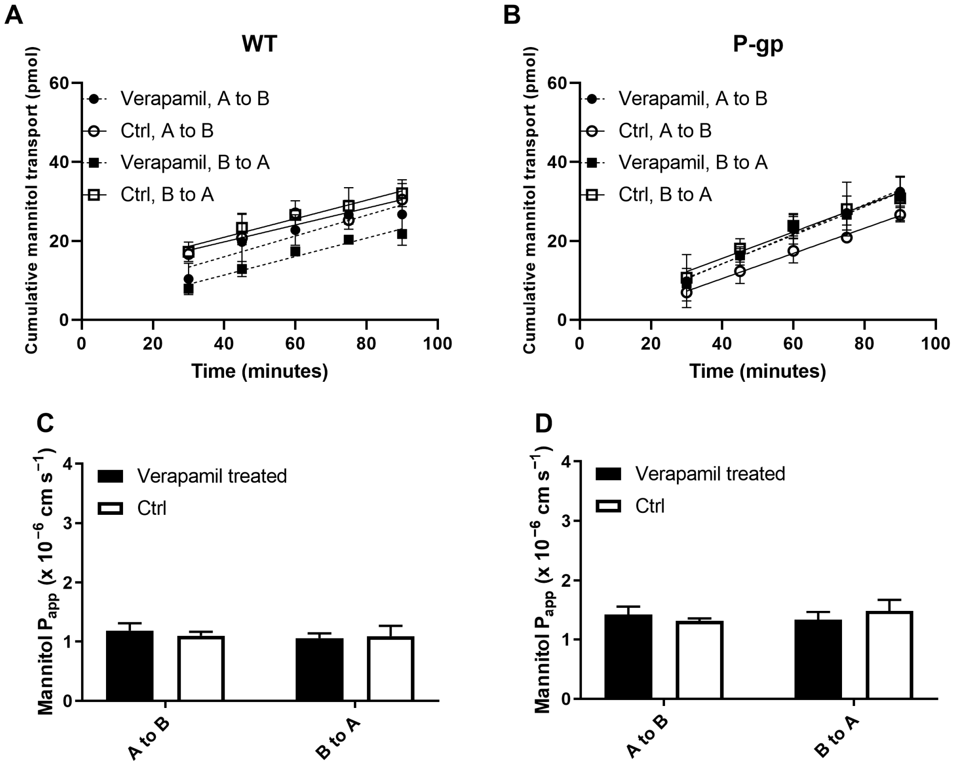

Figure S1. Mannitol bi-directional transport across MDCKII WT (A) and P-gp (B) cell monolayers with and without the transporter inhibitor, verapamil; P_{app} values obtained from the transport assays (C & D). Data represented as mean±SD of three biological replicates.

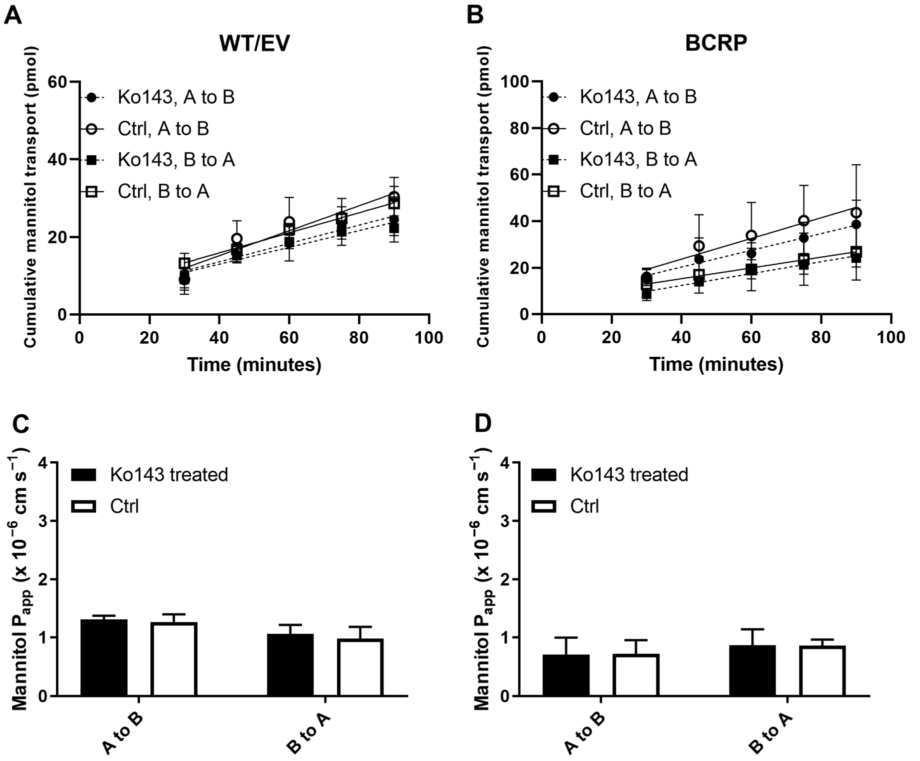

Figure S2. Mannitol bi-directional transport across MDCKII WT/EV (A) and BCRP (B) cell monolayers with and without transporter inhibitors (Ko143 and Ctrl); P_{app} values obtained from the transport assays (C & D). Data represented as mean±SD of three biological replicates.

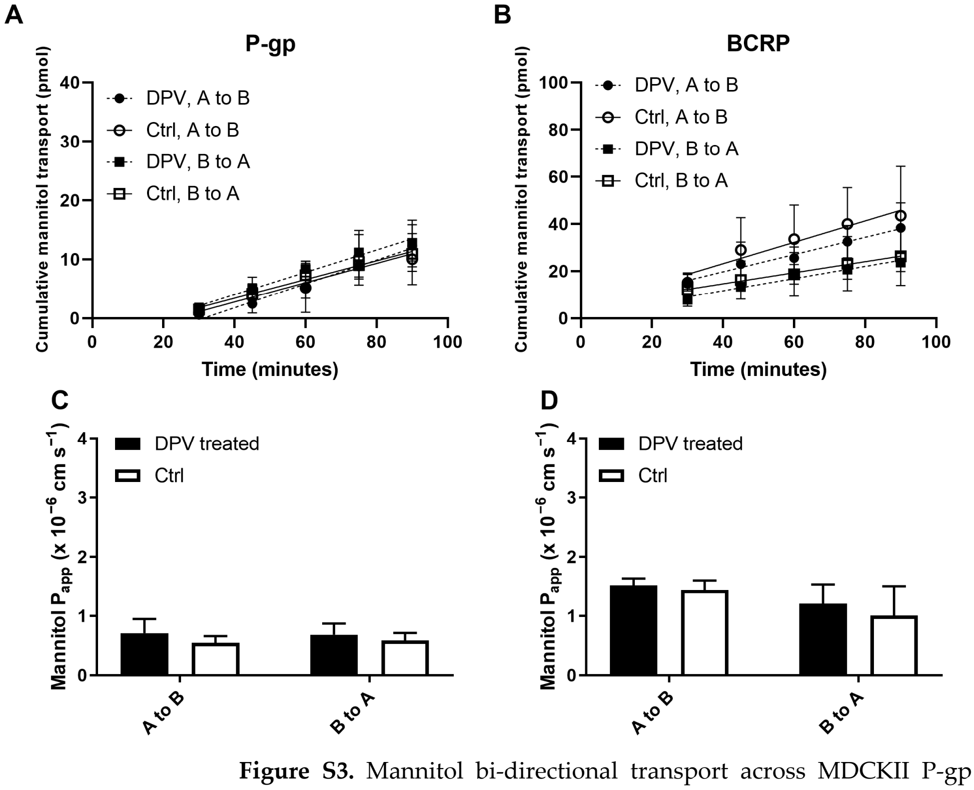

Figure S3. Mannitol bi-directional transport across MDCKII P-gp (A) and BCRP (B) cell monolayers with and without DPV (DPV and Ctrl); P_{app} values obtained from the transport assays (C to D). Data represented as mean±SD of three biological replicates.

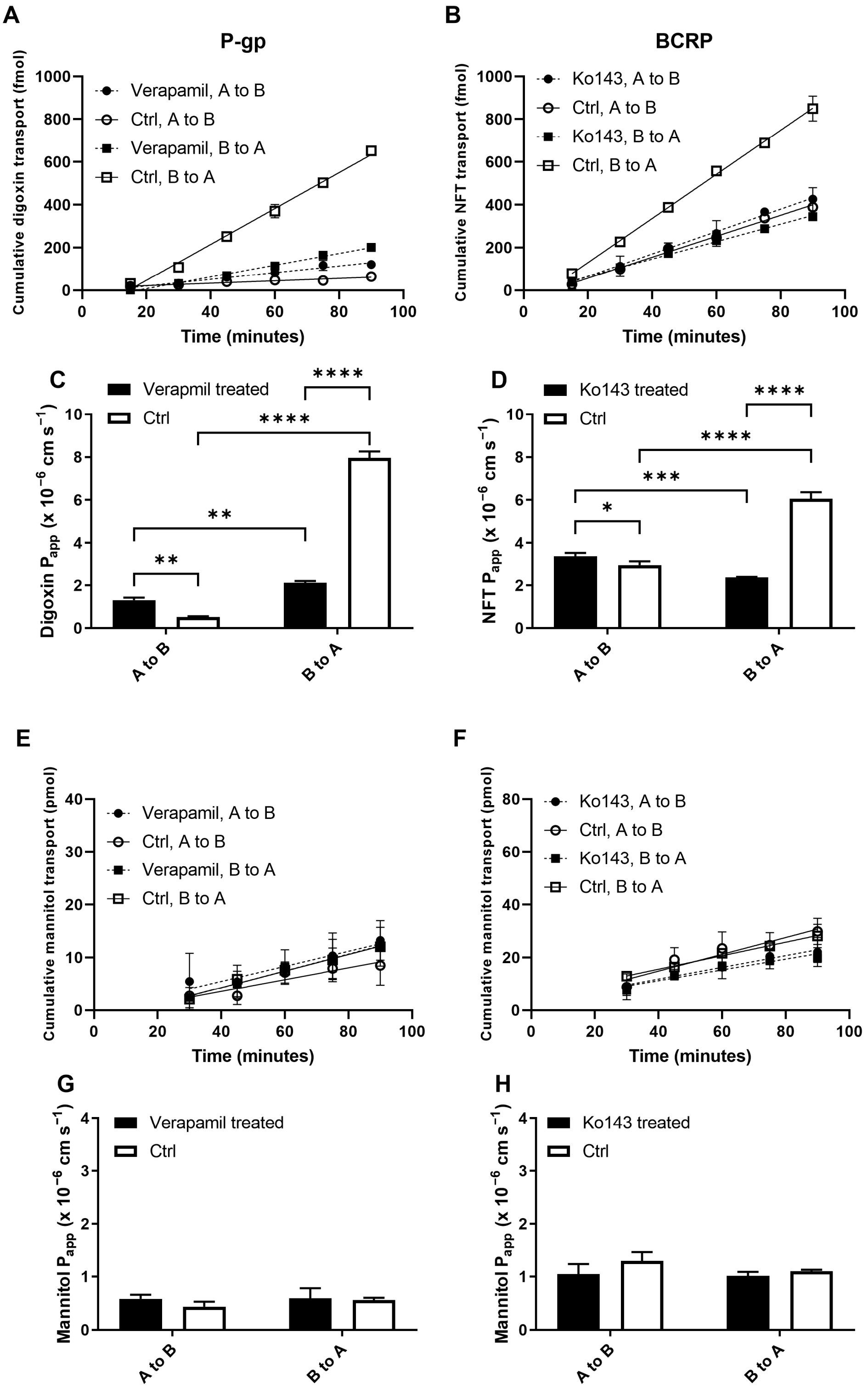

Figure S4. Bi-directional transport of digoxin (A) and mannitol (E) across MDCKII P-gp cell monolayers with and without the P-gp inhibitor, verapamil; Bi-directional transport of nitrofurantoin (B) and mannitol (F) across MDCKII BCRP cell monolayers with and without the BCRP inhibitor, Ko143; P_{app} values obtained from the transport assays (C, D, G & H). Data represented as mean±SD of three biological replicates. * $p < 0.05$. ** $p < 0.01$. *** $p < 0.001$. **** $p < 0.0001$ (Student's *t*-test).
